# The association between oral inflammatory load and semen and sperm functional analysis: A cross-sectional study

**DOI:** 10.34172/joddd.2023.37106

**Published:** 2023-11-11

**Authors:** Reza Pourabbas, Samin Farajzadeh, Amirreza Babaloo, Azar Pazhohan, Mehrnoosh Sadighi, Sakineh Hajebrahimi, Sajjad Pourabbas, Howard C Tenenbaum

**Affiliations:** ^1^Department of Periodontology, Faculty of Dentistry, Tabriz University of Medical Sciences, Tabriz, Iran; ^2^Dental and Periodontal Research Center, Faculty of Dentistry, Tabriz University of Medical Sciences, Tabriz, Iran; ^3^Omid Infertility Treatment Center, Valiasr Hospital, Tabriz, Iran; ^4^Research Center for Evidence-based Medicine, Urology Department, Faculty of Medicine, Tabriz University of Medical Sciences, Tabriz, Iran; ^5^School of Kinesiology and Health Sciences, York University, Toronto, Canada; ^6^Faculty of Dentistry, University of Toronto, Toronto, Canada; ^7^Faculty of Medicine, University of Toronto, Toronto, Canada

**Keywords:** Infertility, Inflammation, Male, Neutrophils, Periodontitis, Spermatozoa

## Abstract

**Background.:**

Studies have suggested a correlation between periodontitis and reduced male fertility. Inflammation has been described as the link between these ailments. Oral inflammatory load (OIL) can be measured through oral polymorphonuclear neutrophil (oPMN) count, which is associated with periodontal diseases. This cross-sectional study assessed the possible correlation between OIL and the functional parameters of sperm cells.

**Methods.:**

In 229 volunteers, oral rinse and semen samples were assessed for oPMN, semen polymorphonuclears (sPMNs), sperm concentration, total sperm count, motility, morphology, and sperm DNA fragmentation index (SDFi). A multiple linear regression model was conducted to evaluate the relationships between oPMN and semen parameters.

**Results.:**

The effect of elevated oPMN counts on total motility rate, progressive rate, and percentage of sperm cells with normal morphology was significant (*P*<0.001), with an inverse relationship, i.e., with every unit increase in oPMN count, the mentioned parameters would decline by 0.573, 0.367, and 0.407 units, respectively. oPMN counts also correlated positively with sPMN counts and SDFi (*P*<0.001), i.e., with every unit increase in the oPMN measures, sPMN counts would increase by 0.126 million/mL, with an 0.733% increase in SDFi. However, there was no significant association between oPMN counts and sperm concentration.

**Conclusion.:**

OIL, as represented by oPMN counts, might affect male fertility as there is a positive correlation between the levels of these inflammatory cells and decreased sperm motility, abnormal morphological changes, increased sPMN counts, and increased SDFi.

## Introduction

 Infertility affects approximately 15% of couples worldwide.^1^ Male factor infertility is attributed to 20%‒70% of infertile couples.^2^ To evaluate the clinical status of male fertility, conventional semen analysis is conducted, and it includes an assessment of volume, sperm concentration, total sperm count, total motility, progressive motility, vitality, pH, and morphology.^3,4^ However, the results of semen analysis do not consider the diverse biological properties of the spermatozoon as a highly specialized cell. Therefore, these types of analyses alone are only moderately predictive of male fertility.^5,6^ In the past few years, there has been a growing interest in sperm DNA integrity and how it impacts male fertility.^7^ The extent of DNA damage/fragmentation in sperm samples is known as the sperm DNA fragmentation index (SDFi) and is reported as the percentage of sperm DNA that has been damaged.^8^ Although the exact mechanisms by which DNA damage/fragmentation occurs in spermatozoa are not understood thoroughly, various pathological conditions such as varicocele, infection, leukocytospermia (high concentrations of leukocytes in semen samples), environmental (e.g., excessive heat), and even occupational stress can disturb the highly refined biochemical events that ensure the integrity of sperm DNA, ultimately leading to abnormal chromatin structure that is incompatible with fertility.^6,9,10^

 Other factors can also contribute to leukocytospermia, such as smoking.^11^ Furthermore, leukocytes, especially polymorphonuclear neutrophils (PMNs), generate molecules such as hydrogen peroxide, known as reactive oxygen species (ROS), leading to oxidative stress, which can cause cellular damage and, in particular, damage to DNA as seen by breaks in DNA strands within spermatozoa. Therefore, leukocytospermia and sperm DNA damage are interlinked and can be detrimental to clinical fertility status.^12^

 Neutrophils are an essential component of the innate immune system. These leukocytes are abundant in the bloodstream and are recruited to various tissues at sites of inflammation, particularly when microbial invasion is also present.^13^

 The oral cavity is lined with a mucosal barrier. However, it is still exposed to large amounts of microbial biofilm; thus, even when there are no clinical signs of inflammation, neutrophils are constantly recruited to the oral cavity and enter the saliva mainly through the gingival crevicular fluid to form a surveillance system.^14^ These particular neutrophils have been termed “para-inflammatory.”^15-17^ Inflammatory reactions, e.g., periodontitis and gingivitis, increase oral neutrophil counts.^18,19^ Concurrent with increased numbers of oral polymorphonuclear neutrophils (oPMNs) associated with periodontitis, phenotypic shifts of these cells have also been demonstrated. Some phenotypes have been classified as being “proinflammatory” leukocytes.^15,17,20^

 It has been suggested that oPMN counts can be characterized as being representative of ‘oral inflammatory load’ (OIL).^19^ Past research by our collaborators suggests that a 30-second oral rinse is sufficient to collect measurable numbers of oral neutrophils to assess and quantify OIL.^19,21^

 Several studies have investigated the association between OIL and systemic diseases or medical conditions concerning other human body organs.^22-25^

 Concerning the primary objectives of this investigation, we note that other studies have assessed the correlation between conventional semen parameters and conventional clinical gingival health parameters, including pocket depth, clinical attachment loss, and bleeding on probing.^26,27^ The findings are compelling but require further validation. This study investigated the association between OIL, assessed by measures of oPMN counts, and several conventional and more novel parameters of seminal health and function, including sperm counts, sperm motility, morphology, and SDFi and seminal polymorphonuclear neutrophil (sPMN) concentrations.

## Methods

 This cross-sectional study was conducted in accordance with the Declaration of Helsinki of 1964, as revised in 2013.

###  Study population and patient selection

 To obtain an accurate sample size, a pilot study was conducted and according to r = 0.6 between the variables oPMN counts and SDFi using software PS (PS: power and sample size calculation version 3.1 for Microsoft Windows Vista, by William D. Dupont and Walton D. Plummer, Jr.) with α = 0.05 and 90% power, a sample size of 173 participants was estimated. To compensate for possible errors in providing standard semen samples and unsuitable results, e.g., azo- or severe oligospermia, failure in sample collection and/or other technical problems, the primary sample size was increased to 235.

 The participants were recruited from a private infertility clinic from July to October 2022. Written and verbal informed consent were obtained prior to their enrollment.

###  Eligibility criteria

 The men aged 20‒60 referred to the infertility clinic were invited to participate in the study. Inclusion criteria were based on the following: having a systemic disease-free medical history. Factors such as a history of diabetes, heart disease, hypertension, hyperthyroidism/hypothyroidism, anemia, serious infections, hepatitis, and immuno-compromised states were considered exclusionary. In addition, participants were excluded from the study if there was evidence of mucocutaneous lesions or any suspicious rashes or ulcers in the oral cavity; abscesses, fistulas, and rampant caries were also considered exclusion factors. Concerning reproductive health factors, patients with a history of varicocele, varicocelectomy, or any testicular abnormalities and obstructive disorders in the reproductive system were excluded. Patients who had taken antibiotics or steroids within the last 30 days and patients who worked or lived in environments with high temperatures or who worked with toxic substances such as pesticides, lead, or other chemicals found in rubber and plastic utilized in food and drink containers, heavy smokers and drinkers were also excluded due to the adverse effects of these substances on SDFi and sperm quality.^7^ However, according to the information provided by the participants in the questionnaire, light or moderate smokers and light/social alcohol consumers were included.

###  Questionnaires

 A self-administered structured questionnaire was distributed among all participants before providing samples. Information on the sociodemographic background (age, body weight, height, place of residence, level of education, and occupation), lifestyle habits (smoking and alcohol consumption), oral hygiene habits (frequency of brushing, dental floss usage, presence or absence of gingival bleeding during brushing) and general health (chronic diseases and medication use) was collected.

###  Oral rinse sample collection

 The patients were asked not to eat or drink for at least 30 minutes prior to providing samples. Each participant was given a centrifuge tube containing 10 mL of 0.9% saline solution and was instructed to rinse for 30 seconds and expectorate into the centrifuge tube afterward. All the samples were processed immediately after collection. Although this collection method deviates somewhat from that described previously,^21^ the investigators believed that determining OIL was not hindered.

 Rinse samples were then centrifuged at 2500 rpm for 5 minutes. Afterward, 8 mL of the supernatant liquid was discarded, leaving only 2 mL of the sample containing the pellet and a small quantity of liquid. The pellet was then resuspended in the liquid by stirring the tube gently.

###  Assessment of oPMN levels

 Myeloperoxidase is an enzyme found predominantly in neutrophils and is largely stored in cytoplasmic granules, and as such, it is a very useful marker for identifying neutrophils.^28^

 Myeloperoxidase-stained neutrophils were detected according to the following protocol:

 The working solution was prepared using the reagents below:

1. Phosphate-buffered saline, 67 mmol/L, pH = 6.0
2. Saturated ammonium chloride (NH_4_Cl) solution
3. Disodium ethylenediamine tetra-acetic acid (Na_2_EDTA), 148 mmol/L
4. Substrate: dissolve 2.5 mg of *o*-toluidine in 10 mL of 0.9% (9 g/L) saline
5. Hydrogen peroxide (H_2_O_2_), 30% (v/v)

To prepare the working solution, 1 mL of saturated NH_4_Cl solution, 1 mL of 148 mmol/L Na_2_EDTA, and 10 µL of 30% (v/v) H_2_O_2_ were added to 9 mL of o-toluidine substrate and mixed well. This solution can be used up to 24 hours after preparation.^4^

 Two sample aliquots were removed, each containing 0.1 mL, mixed separately with 0.9 mL of previously made working solution, and incubated at room temperature for 20‒30 minutes.

 We then proceeded to load each side of the hemocytometer with the prepared samples.

 The chambers were examined with phase-contrast optics at × 200 magnification. We observed that myeloperoxidase-positive oPMNs retained their round shape and stained a brown color, and were readily distinguished from myeloperoxidase-negative cells.

 Stained cells were counted and processed according to hemocytometer user instructions, and afterward, an average concentration was reported as the final oPMN count.

###  Semen collection and analysis

 The semen samples were collected and analyzed according to WHO 2010 laboratory manual for examining and processing human semen in a medical setting. In addition, Diff-Quik staining technique was used to evaluate sperm cell morphology. The methodology employed to evaluate SDFi was the sperm chromatin dispersion test using light microscopy.^4^ The participants were asked to remain abstinent for 3‒5 days. Semen samples were collected in sterile plastic containers, according to the protocol of the infertility center and analyzed.

###  Statistical analysis

 The results of the descriptive analysis were reported by frequencies and percentages for qualitative variables. Indices of mean and standard deviation, minimum and maximum values were utilized for quantitative variables with normal distribution. As for the skewed variables, median, first and third interquartiles, and minimum and maximum values were utilized. Pearson’s correlation coefficient was used to investigate the relationship between the two quantitative variables if normality was evident, and Spearman’s correlation coefficient was used if the normal distribution was not present. Student’s independent t-test was used to evaluate the differences between the means of quantitative variables between the two different groups, such as smokers and non-smokers. Simple linear regression was employed to investigate the impact of each independent variable on oPMN counts. Also, simple linear regression analysis was used to investigate the relationship between and the effect of each independent variable on the dependent variables. Furthermore, the variables with significance at 0.2 level in univariate regression were put into a multiple linear regression model for additional analysis. Statistical significance was set at *P* < 0.05. Statistical analysis was performed using SPSS (IBM SPSS Statistics for Windows, version 26.0.0, IBM Corp., Armonk, N.Y., USA).

## Results

**Figure 1:**
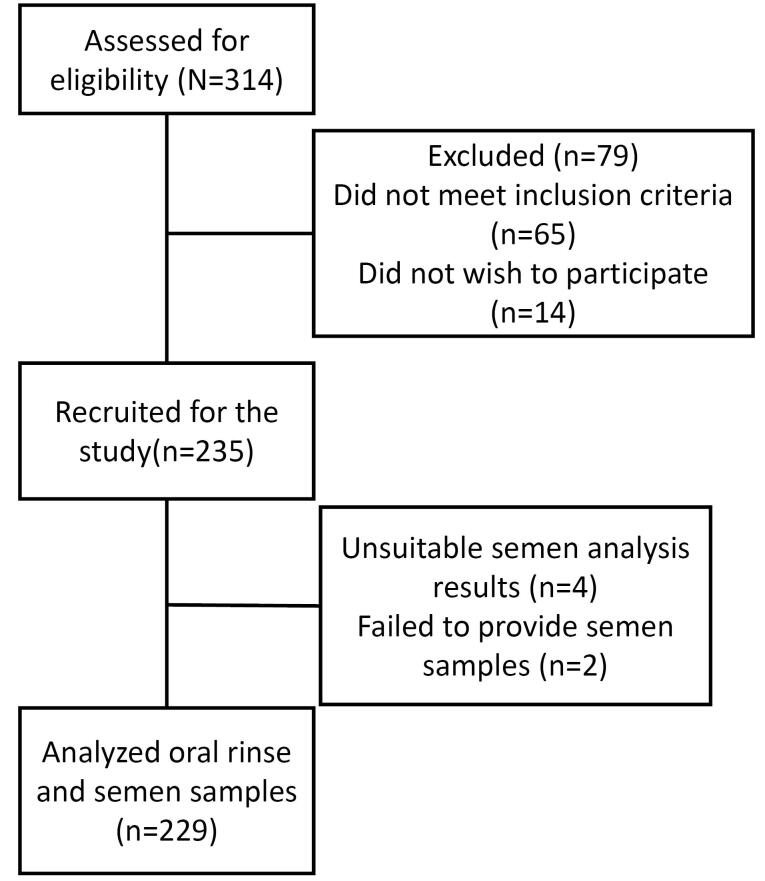
Study flowchart diagram

 The data and results of 229 men were analyzed. Six participants were excluded due to sampling failure and/or technical problems (Figure 1).

###  Sociodemographic information

 The mean age of participants was 39.50 ± 6.18 (range: 28‒56) years. The mean BMI was 27.78 ± 2.17 (range: 22.49‒33.41) kg/m^2^. Light/moderate smoking and light/social drinking rates were 65.5% and 56.8%, respectively.

###  Laboratory results

 The mean concentration of oPMN was 20.62 ± 9.85 (range: 5.00‒39.80) × 10^5^/mL.

 The semen parameters of participants are presented in Table 1. The results showed no statistically significant association between oPMN counts and sperm concentration (*P* = 0.406). However, there was a small but positive relationship between oPMN and total sperm count (ρ = 0.164, *P* = 0.013).

**Table 1:** Semen parameters of participants

**Variables**	**Mean±SD or Median (Q1, Q3)**^a^	**Min**	**Max**
Semen volume (mL)	4.145 ± 1.24	1.5	7.0
Sperm concentration (million/mL)	66.26 ± 36.728	5	135
Total count (million)	240.00 (105.60, 405.00)	17.50	660.00
Progressive rate (%)	15.00 (5.00,30.00)	0	40
Non-progressive rate (%)	18.55 ± 8.153	5	40
Total motility (%)	35.26 ± 18.22	5.00	70.00
Sperm cells with normal morphology (%)	4 (2.40,8.40)	.0	19.4
sPMN concentration (million/mL)	2 (1,5)	.0	9.0
SDFi (%)	22.40 ± 9.72	6.3	42.1

SD, Standard Deviation; Q, Quartile; Min, Minimum; Max, Maximum; sPMN, seminal polymorphonuclear neutrophil; SDFi, Sperm DNA Fragmentation index. ^a^Mean and standard error are reported for quantitative parameters with normal distribution, and for those parameters not conforming to a normal distribution median, first and third quartile are reported.

 An inverse correlation was found between oPMN and total motility rate (ρ = -0.341, *P* < 0.001). The same result was observed between oPMN and progressive rate findings (ρ = -0.320, *P* < 0.001).

 A negative correlation was noted between oPMN and the percentage of sperm cells with normal morphology (ρ = -0.775, *P* < 0.001).

 The correlation between oPMN and sPMN counts was statistically significant and positively related (ρ = 0.669, *P* < 0.001). A strong positive correlation was also found between oPMN and SDFi (ρ = 0.826, *P* < 0.001).

 There was no significant correlation between BMI and oPMN counts, but there did appear to be a negative, albeit small, correlation with age (BMI; *P* = 0.511; Age ρ = -0.155, *P* = 0.019).

 The data showed significant relationships between oPMN, smoking, and alcohol consumption. The average oPMN counts in non-smoking participants were 8.1 units lower than current smokers (B = -8.100, *P* < 0.001). Along the same lines, the oPMN levels in participants who did not consume alcohol were 3.3 units lower than those who did (B = -3.332, *P* = 0.008).

 Simple linear regression results for different dependent variables concerning sperm parameters are demonstrated in Table 2.

**Table 2:** Simple linear regression results with different dependent variables

**Variables**	**Sperm concentration** **(million/mL)**	**Total count** **(million)**	**Progressive rate** **(%)**	**Total motility** **(%)**	**sPMN concentration** **(million/mL)**	**Normal morphology** **(%)**	**SDFi** **(%)**
Age (y)							
Coefficient	-0.343	-0.183	-0.082	-0.102	-0.044	0.039	-0.349
SE	0.394	1.733	0.132	0.196	0.023	0.057	0.102
*P* value	0.385	0.916	0.537	0.602	0.057	0.498	0.001
BMI (kg/m^2^)							
Coefficient	0.317	-1.427	0.206	0.180	-0.082	-0.160	0.123
SE	1.125	4.942	0.378	0.558	0.066	0.162	0.298
*P* value	0.779	0.773	0.587	0.747	0.213	0.325	0.680
Smoking							
Coefficient	-12.529	-30.347	3.062	3.446	-1.294	2.974	-8.655
SE	5.049	22.392	1.708	2.529	0.288	0.713	1.227
*P* value	0.014	0.177	0.074	0.174	< 0.001	< 0.001	< 0.001
Alcohol							
Coefficient	3.176	18.805	8.0809	14.663	-0.686	0.913	-4.575
SE	4.905	21.537	1.543	2.233	0.285	0.707	1.264
*P* value	0.518	0.384	< 0.001	< 0.001	0.017	0.198	< 0.001
oPMN ( × 10^5^/mL)							
Coefficient	0.206	1.785	-0.418	-0.631	0.133	-0.389	0.815
SE	0.274	1.080	0.078	0.115	0.012	0.025	0.037
*P* value	0.406	0.100	< 0.001	< 0.001	< 0.001	< 0.001	< 0.001

SE, Standard Error; SDFi, Sperm DNA Fragmentation index; oPMN, Oral polymorphonuclear neutrophils.

 To investigate the significance and impact of each independent variable on dependent sperm variables further, the variables with significance at the level of 0.2 based on univariate regression were put into the multiple linear regression model, as seen in Table 3. After adjusting for confounding factors, the following results were obtained:

**Table 3:** Multiple linear regression model for different dependent variables concerning semen parameters

	**Unstandardized coefficient, B**	**95% confidence interval **		***P* value**
		**Lower bound**	**Upper bound**	
For total count (million)				
Smoking	-18.040	-66.594	30.514	0.465
oPMN( × 10^5^/mL)	1.420	-0.928	3.767	0.235
For the progressive rate (%)				
Smoking	-0.515	-3.888	2.859	0.764
Alcohol	6.916	3.746	10.087	< 0.001
oPMN ( × 10^5^/mL)	-0.367	-0.536	-0.198	< 0.001
For total motility (%)				
Smoking	-2.187	-7.051	2.677	0.376
Alcohol	11.660	7.089	16.232	< 0.001
oPMN ( × 10^5^/mL)	-0.573	-0.817	-0.329	< 0.001
For sPMN concentration (million/mL)				
Age	-0.008	-0.045	0.029	0.661
Smoking	-0.427	-1.076	0.221	0.195
Alcohol	-0.180	-0.644	0.285	0.447
oPMN ( × 10^5^/mL)	0.126	0.100	0.152	< 0.001
For normal morphology (%)				
Smoking	-0.489	-1.875	0.896	0.487
Alcohol	-0.719	-1.771	0.334	0.180
oPMN ( × 10^5^/mL)	-0.407	-0.463	-0.351	< 0.001
For SDFi (%)				
Age	-0.128	-0.245	-0.012	0.031
Smoking	-2.272	-4.278	-0.265	0.027
Alcohol	-1.839	-3.365	-0.314	0.018
oPMN ( × 10^5^/mL)	0.733	0.651	0.814	< 0.001

B, unstandardized beta; oPMN, oral polymorphonuclear neutrophils; sPMN, seminal polymorphonuclear neutrophils; SDFi, Sperm DNA Fragmentation index.

 Light/social consumption of alcohol and oPMN counts significantly affected sperm progressive rate. Followed by adjustment for confounding factors, the progressive rate in participants who did not consume alcohol was, on average, 6.916% higher than those who did (B = 6.916, *P* < 0.001), and with every one-unit increase in oPMN, the progressive rate would decline by an average of 0.367% (B = -0.367, *P* < 0.001).

 Accordingly, alcohol consumption and the degree of OIL based on oPMN counts had significant effects on total motility, such that, on average, total motility was 11.66% higher in participants who did not drink alcohol (B = 11.660, *P* < 0.001). Also, with every one-unit rise in oPMN, the total motility rate would decline by an average of 0.573% (B = -0.573, *P* < 0.001).

 Seminal polymorphonuclear concentration was affected by OIL in that, on average, for every one-unit rise in oPMN counts, the sPMN levels increased by 0.126 units or 126000 cells/mL (B = 0.126, *P* < 0.001).

 With every single-unit rise in oPMN, the percentage of sperm cells with normal morphology decreased by an average of 0.407% (B = -0.407, *P* < 0.001).

 Overall, the findings demonstrated that age, smoking, alcohol consumption, and oPMN levels had significant effects on SDFi. In this regard, with every 1-year increase in age, SDFi would decline by 0.128 units (B = -0.128, *P* = 0.031), and with every one-unit increase in oPMN, SDFi would increase by 0.733% (B = 0.733, *P* < 0.001). Also, SDFi in non-smoking participants and participants who did not consume alcohol was lower by 2.272% and 1.839%, respectively, in comparison to smoking participants and those who did consume alcohol (B = -2.272, *P* = 0.027) (B = -1.839, *P* = 0.018).

## Discussion

 Infertility and periodontal disease are multifactorial ailments. Studies investigating the association between oral health and male factor infertility are scarce, often with contradictory findings. Despite this, a systematic review published in 2018 still demonstrated a positive association between chronic periodontitis and male factor infertility based on the assessment of 6 out of 7 included studies.^27^

 While using clinical parameters such as bleeding on probing, pocket depth, and clinical attachment loss remains a routine and conventional method to detect the periodontal status in a dental setting, these measurements are rarely available in most medical laboratories involved with sperm analysis. Furthermore, it has been suggested that when studying the relationship between oral and non-oral (systemic and/or localized) diseases or conditions, oral inflammation should be evaluated rather than a specific diagnosed disease, such as periodontitis.^22^ Furthermore, loss of attachment and probing depths can be considered indicators of past disease but do not necessarily correlate with the presence of active or ongoing periodontal disease, while the increased levels of OIL are associated strongly with ongoing disease.^29^

 Employing OIL using oPMN counts is a simple, reproducible, and noninvasive method, and the correlation between oPMN levels and the severity of periodontal disease has been validated by a previous study.^19^ This can also be assessed in a non-dental setting, which permits the study of relationships between oral diseases like periodontitis and non-oral diseases. However, it is necessary to note that oral inflammation can arise from a wide range of diseases, including but not limited to mucocutaneous disease. This is noteworthy because it is probable that lesions arising from a mucocutaneous disease will also contribute to the OIL, which requires further studies since, from a medical perspective, the source of OIL increases might be less important than the presence of increased OIL regardless of the source. However, upon identifying increased OIL levels, a medical referral to a dentist or periodontist can be made. Hence, readily measured OIL, based on oPMN counts, can be used as a screening test for oral inflammation levels, and of course, further investigations are necessary to reach an accurate diagnosis.^19,21,22,30,31^

 Several studies have measured OIL using different techniques, including flow cytometry, ABTS (2,2’-azino-bis-3-ethylbenzothiazoline-6-sulphonic acid), and cell staining with acridine orange.^19,20,25^ In this study, we used a histochemical method called the “peroxidase test,” also known as the “myeloperoxidase test,” to count oral neutrophils.^32^ This procedure employs ortho-toluidine for cellular peroxidase staining purposes and is currently being employed in research and clinical practice in other spheres of medicine.^4^ However, to the best of our knowledge, there is no report of using this technique to evaluate oPMNs.

 The present study found a negative association between oPMN counts and sperm motility parameters (progressive rate and total motility), suggesting that the higher the oPMN levels, the lower the percentage of motile sperm cells. However, there was no significant association between oPMN levels and sperm concentration. These findings are consistent with those of previous studies.^26,33^ Nwhator et al^34^ found a significant association between periodontitis and subnormal sperm counts only among the 33‒38 age group. However, they did find a significant association between poor oral hygiene and subnormal sperm count levels. This impact of sperm motility on male fertility is large and has to be considered when assessing for problems related to it.

 This study found a significant and strong positive correlation between oPMN and sPMN levels. Neutrophils in the semen produce ROS, which can harm sperm cells, impairing the motility, morphology, and chromatin package of spermatozoa. With high semen neutrophil counts, motility and percentage of sperm cells with normal morphology decrease while SDFi increases.^12^

 The mechanism by which oral inflammation and periodontal condition can be related to male reproductive status has always been debated.

 While some studies have found a similar association between oral bacterial flora and bacteriospermia and have suggested that oral pathogens can penetrate through the epithelial barrier and enter the circulatory system, leading to bacteremia and bacteriospermia,^35^ others have suggested that certain periodontal pathogens can indirectly contribute to increased serum levels of proinflammatory cytokines such as IFN-γ and TNF-α. Therefore, the presence of these pathogens in the oral cavity can lead to an increased risk of systemic diseases.^36^

 The role of microbial elements of periodontal disease or other oral cavity infections as risk factors/indicators in other systemic diseases, such as cardiovascular diseases, is well-documented.^37-39^ For instance, the role of matrix metalloproteinases (MMPs), particularly MMP-7 and, to a lesser extent, MMP-3, in the breakdown of fetuin, which is an anti-inflammatory protein, has been addressed. Increased MMPs in inflammatory diseases such as periodontitis and the resultant destruction of fetuin potentially upregulate the risk of non-oral diseases.^40^ In any case, in the study reported here, microbial factors were not assessed, as the main focus was on oral and reproductive inflammation.

 Also, considering that the participants of this study were selected from male partners of couples referred to the infertility clinic, lacking in generalizability may be a reasonable limitation concerning the study design reported here.

 While the current study found a significant correlation between oral inflammation and seminal parameters, further investigation is needed to address the association between high levels of oral inflammation as a risk factor for male infertility. Assessing clinical periodontal parameters along with oPMN for confirming the periodontal status of the participants is also recommended, particularly if an assessment of OIL is not possible in any one setting.

## Conclusion

 According to the findings of this study, high OIL is correlated with adverse effects on parameters of male reproductive capacity. The participants with higher oPMN counts were found to have diminished sperm and semen qualities such as sub-motility, lower than sufficient sperm morphology, higher rates of semen neutrophil counts, and higher scores for SDFi.

## Competing Interests

 The authors declare no competing interests.

## Ethical Approval

 Written and verbal informed consent was obtained prior to the participants’ enrollment.

 This cross-sectional study was approved by the Research Ethics Committee of Tabriz University of Medical Sciences under the code IR.TBZMED.REC.1401.331.

## References

[1] Sharlip ID, Jarow JP, Belker AM, Lipshultz LI, Sigman M, Thomas AJ (2002). Best practice policies for male infertility. Fertil Steril.

[2] Agarwal A, Mulgund A, Hamada A, Chyatte MR (2015). A unique view on male infertility around the globe. Reprod Biol Endocrinol.

[3] Cooper TG, Noonan E, von Eckardstein S, Auger J, Baker HW, Behre HM (2010). World Health Organization reference values for human semen characteristics. Hum Reprod Update.

[4] World Health Organization (WHO). WHO Laboratory Manual for the Examination and Processing of Human Semen. WHO; 2010.

[5] Zini A, Kamal K, Phang D, Willis J, Jarvi K (2001). Biologic variability of sperm DNA denaturation in infertile men. Urology.

[6] Evenson DP, Larson KL, Jost LK (2002). Sperm chromatin structure assay: its clinical use for detecting sperm DNA fragmentation in male infertility and comparisons with other techniques. J Androl.

[7] Agarwal A, Majzoub A, Baskaran S, Panner Selvam MK, Cho CL, Henkel R (2020). Sperm DNA fragmentation: a new guideline for clinicians. World J Mens Health.

[8] Agarwal A, Said TM (2003). Role of sperm chromatin abnormalities and DNA damage in male infertility. Hum Reprod Update.

[9] Evenson DP, Wixon R (2006). Clinical aspects of sperm DNA fragmentation detection and male infertility. Theriogenology.

[10] Aboulmaouahib S, Madkour A, Kaarouch I, Sefrioui O, Saadani B, Copin H (2018). Impact of alcohol and cigarette smoking consumption in male fertility potential: looks at lipid peroxidation, enzymatic antioxidant activities and sperm DNA damage. Andrologia.

[11] Saleh RA, Agarwal A, Sharma RK, Nelson DR, Thomas AJ Jr (2002). Effect of cigarette smoking on levels of seminal oxidative stress in infertile men: a prospective study. Fertil Steril.

[12] Agarwal A, Said TM (2005). Oxidative stress, DNA damage and apoptosis in male infertility: a clinical approach. BJU Int.

[13] Borregaard N (2010). Neutrophils, from marrow to microbes. Immunity.

[14] Raeste AM, Tapanila T, Tupakka R (1977). Leukocyte migration into the healthy dentulous mouth A study in children, adolescents and adults. J Periodontal Res.

[15] Medzhitov R (2008). Origin and physiological roles of inflammation. Nature.

[16] Hirschfeld J (2019). Neutrophil subsets in periodontal health and disease: a mini review. Front Immunol.

[17] Chadwick JW, Fine N, Khoury W, Tasevski N, Sun CX, Boroumand P (2021). Tissue-specific murine neutrophil activation states in health and inflammation. J Leukoc Biol.

[18] Bhadbhade SJ, Acharya AB, Thakur S (2012). Correlation between probing pocket depth and neutrophil counts in dental plaque, saliva, and gingival crevicular fluid. Quintessence Int.

[19] Landzberg M, Doering H, Aboodi GM, Tenenbaum HC, Glogauer M (2015). Quantifying oral inflammatory load: oral neutrophil counts in periodontal health and disease. J Periodontal Res.

[20] Fine N, Hassanpour S, Borenstein A, Sima C, Oveisi M, Scholey J (2016). Distinct oral neutrophil subsets define health and periodontal disease states. J Dent Res.

[21] Bender JS, Thang H, Glogauer M (2006). Novel rinse assay for the quantification of oral neutrophils and the monitoring of chronic periodontal disease. J Periodontal Res.

[22] Huda S, Doering H, Tenenbaum HC, Whittle W, Sigal MJ, Glogauer M (2015). Oral neutrophil levels: a screening test for oral inflammatory load in pregnancy in a medical setting. J Periodontol.

[23] Wilcox ME, Charbonney E, d’Empaire PP, Duggal A, Pinto R, Javid A (2014). Oral neutrophils are an independent marker of the systemic inflammatory response after cardiac bypass. J Inflamm (Lond).

[24] Cheretakis C, Dror Y, Glogauer M (2005). A noninvasive oral rinse assay to monitor engraftment, neutrophil tissue delivery and susceptibility to infection following HSCT in pediatric patients. Bone Marrow Transplant.

[25] Sharma V, Hanafi A, Overgaard CB, Shaibani M, An K, Dzavik V (2019). Oral inflammatory load in patients with coronary artery disease. J Oral Sci.

[26] Klinger A, Hain B, Yaffe H, Schonberger O (2011). Periodontal status of males attending an in vitro fertilization clinic. J Clin Periodontol.

[27] Kellesarian SV, Yunker M, Malmstrom H, Almas K, Romanos GE, Javed F (2018). Male infertility and dental health status: a systematic review. Am J Mens Health.

[28] Arnhold J, Flemmig J (2010). Human myeloperoxidase in innate and acquired immunity. Arch Biochem Biophys.

[29] Khoury W, Glogauer J, Tenenbaum HC, Glogauer M (2020). Oral inflammatory load: neutrophils as oral health biomarkers. J Periodontal Res.

[30] Moosani A, Sigal MJ, Glogauer M, Lawrence HP, Goldberg M, Tenenbaum HC (2014). Evaluation of periodontal disease and oral inflammatory load in adults with special needs using oral neutrophil quantification. Spec Care Dentist.

[31] Landzberg M. Evaluation of a Colorimetric Assay as a Screening Test for Periodontal Disease. Toronto, ON: University of Toronto; 2009. p. 33-7.

[32] Walker HK, Hall WD, Hurst JW. Clinical Methods: The History, Physical, and Laboratory Examinations. Butterworth-Heinemann; 1990. p. 724. 21250045

[33] Tao DY, Zhu JL, Xie CY, Kuang YP, Chai WR, Lo ECM (2021). Relationship between periodontal disease and male infertility: a case-control study. Oral Dis.

[34] Nwhator SO, Umeizudike KA, Ayanbadejo PO, Opeodu OI, Olamijulo JA, Sorsa T (2014). Another reason for impeccable oral hygiene: oral hygiene-sperm count link. J Contemp Dent Pract.

[35] Bieniek KW, Riedel HH (1993). Bacterial foci in the teeth, oral cavity, and jaw--secondary effects (remote action) of bacterial colonies with respect to bacteriospermia and subfertility in males. Andrologia.

[36] Andrukhov O, Ulm C, Reischl H, Nguyen PQ, Matejka M, Rausch-Fan X (2011). Serum cytokine levels in periodontitis patients in relation to the bacterial load. J Periodontol.

[37] Peters MJ, Symmons DP, McCarey D, Dijkmans BA, Nicola P, Kvien TK (2010). EULAR evidence-based recommendations for cardiovascular risk management in patients with rheumatoid arthritis and other forms of inflammatory arthritis. Ann Rheum Dis.

[38] Wong M, Toh L, Wilson A, Rowley K, Karschimkus C, Prior D (2003). Reduced arterial elasticity in rheumatoid arthritis and the relationship to vascular disease risk factors and inflammation. Arthritis Rheum.

[39] Xiao J, Wang XR, Hu KZ, Li MQ, Chen JW, Ma T (2013). Serum fetuin-A levels are inversely associated with clinical severity in patients with primary knee osteoarthritis. Biomarkers.

[40] Schure R, Costa KD, Rezaei R, Lee W, Laschinger C, Tenenbaum HC (2013). Impact of matrix metalloproteinases on inhibition of mineralization by fetuin. J Periodontal Res.

